# Supplementary material for: Disease-specific loss of microbial cross-feeding interactions in the human gut
Source: Nat Commun. 2023 Oct 20;14:6546. doi: 10.1038/s41467-023-42112-w (PMC10589287; doi:10.1038/s41467-023-42112-w)
Supplement: Supplementary file 1 — Supplementary Information [file 41467_2023_42112_MOESM1_ESM.pdf]

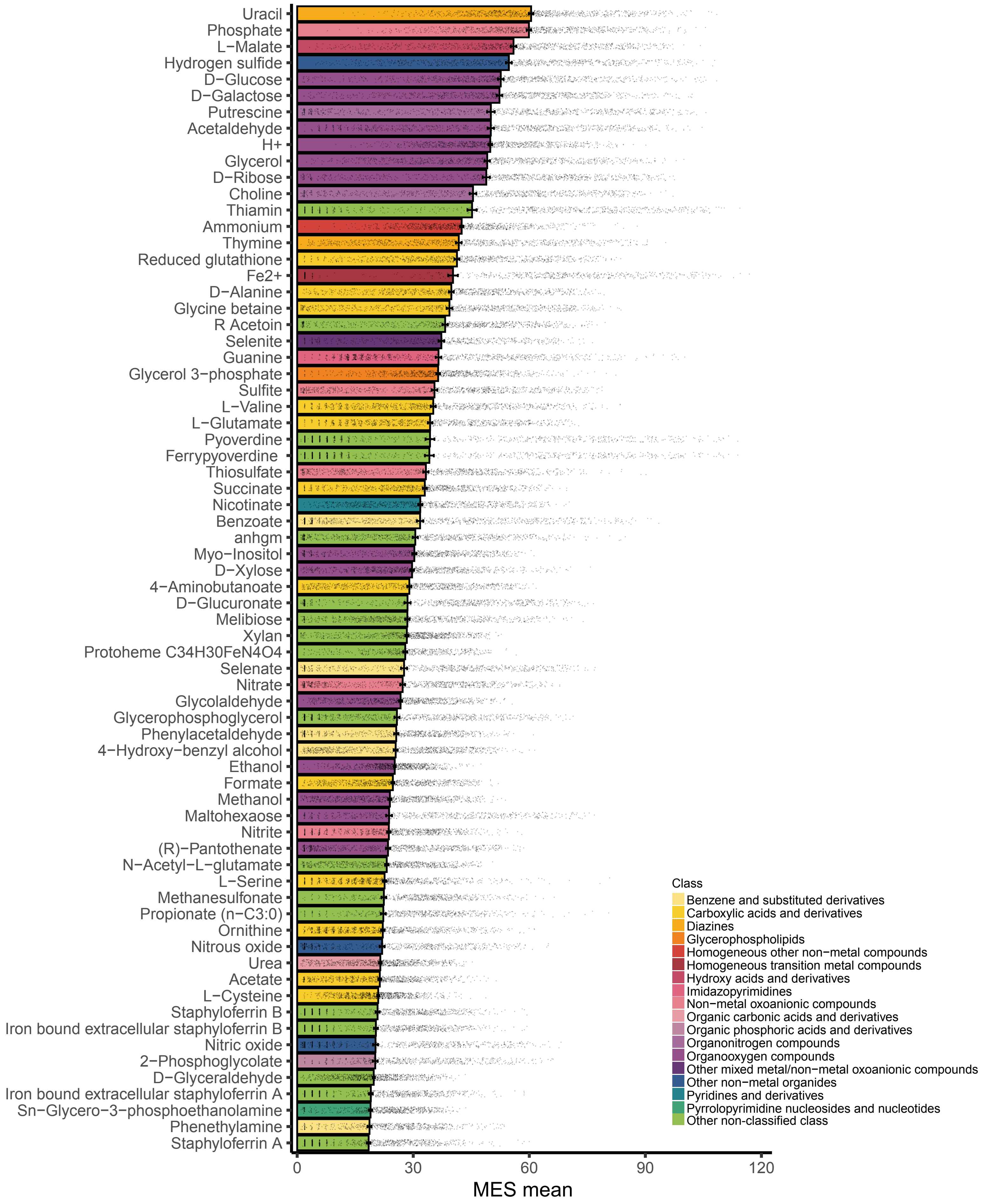

Supplementary Figure S1. Top 70 metabolites with highest Metabolite Exchange Scores (MES) in the healthy human gut microbiome (871 microbiomes). Bars represent standard error, and colors represent metabolite class according to the Human Metabolome Database.

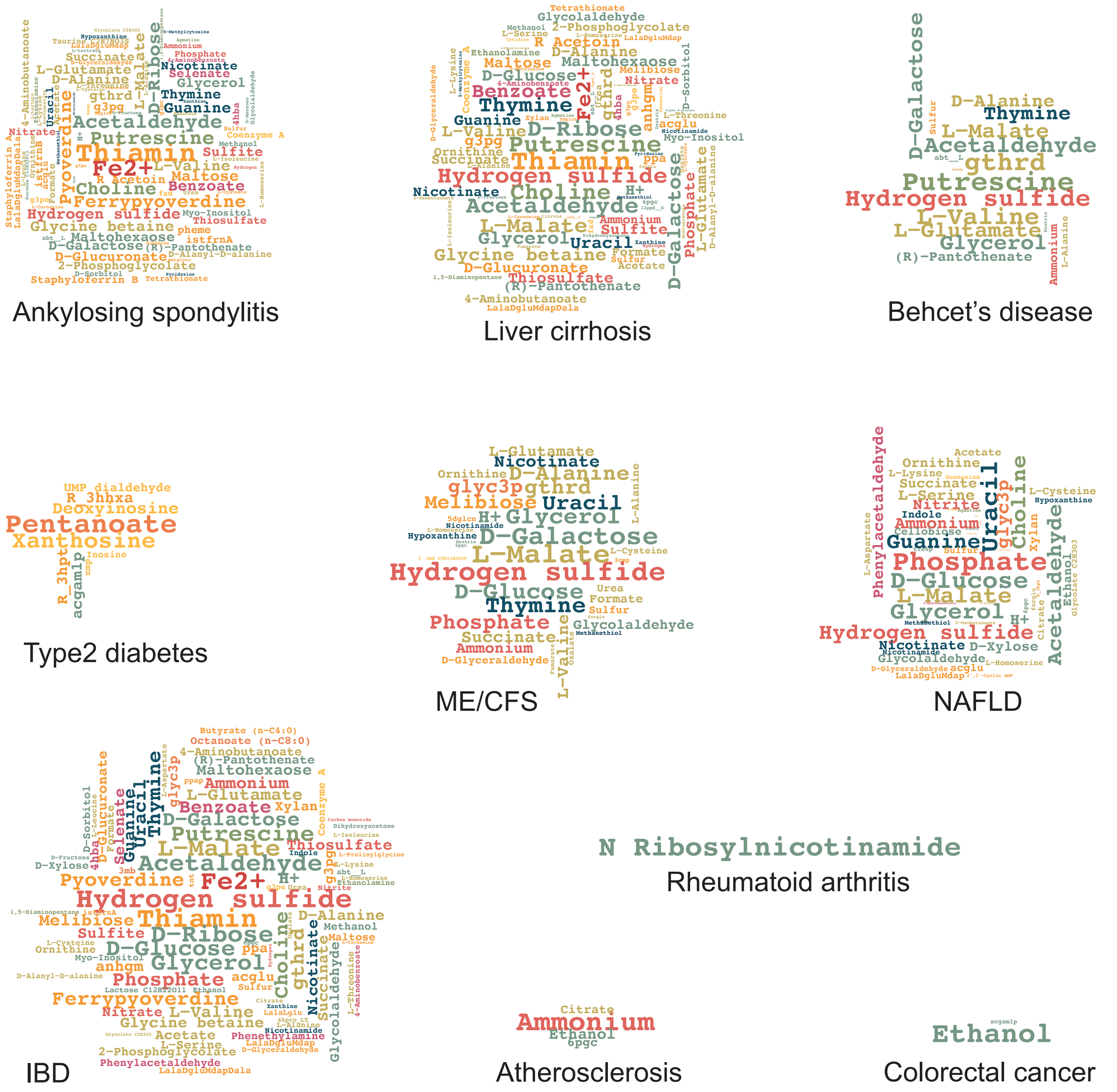

Supplementary Figure S2. Metabolites with significantly reduced Metabolite Exchange Scores (Kruskal Wallis' $p < 0.05$/number of comparisons per category) in the microbiomes associated with 10 disease phenotypes when compared to the healthy group, suggesting significant loss of microbial cross-feeding partners for those metabolites. Letters in word clouds are proportional to the difference in MES between health and disease. While figure 2c (main text) included only the top 5 metabolites with highest MES difference between healthy and diseased groups for each disease (for readability), these word clouds include up to 100 metabolites.
Sample sizes and Bonferroni-corrected p-value thresholds: IBD=inflammatory bowel disease (n=63, p<1.27x10-4), liver cirrhosis (n=54, p<1.30x10-4), Ank=ankylosing spondylitis (n=72, p<1.32x10-4), NAFLD=non-alcoholic fatty liver disease (n=71, p<1.25x10-4), Behcet's disease (n=18, p<2.21x10-4), ME/CSF=myalgic encephalomyelitis/chronic fatigue syndrome (n=17, p<2.99x10-4), T2D=type 2 diabetes (n=32, p<1.37x10-4), Athero=atherosclerosis (n=98, p<1.18x10-4), CRC=colorectal cancer (n=143, p<1.17x10-4), Arthritis=rheumatoid arthritis (n=135, p<1.18x10-4).

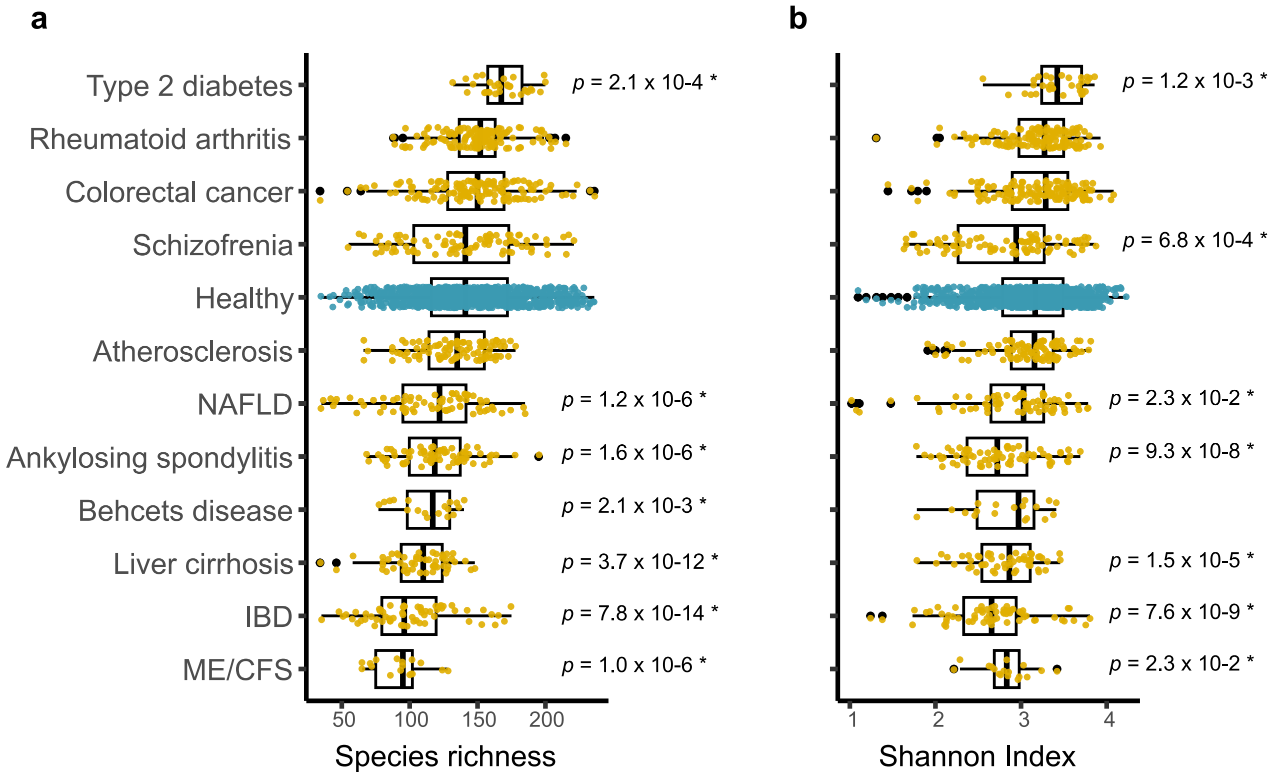

Supplementary Figure S3. Alpha diversity indices per health status, including species richness (a) and Shannon Index (b). Significant *P* values of Wilcoxon test comparing species diversity between healthy and disease categories are shown (corrected for multiple comparisons with Holm). Disease categories and sample sizes: type2 diabetes (n= 32), rheumatoid arthritis (n=135), colorectal cancer (n=143), schizofrenia (n= 87), healthy (n=871), atherosclerosis (n=98), non-alcoholic fatty liver disease (NAFLD, n= 71), ankylosing spondylitis (n=72), Behcets disease (n=18), liver cirrhosis (n=54), inflammatory bowel disease (IBD, n=63), myalgic encephalomyelitis/chronic fatigue syndrome (ME/CFS, n= 17). Box-plot elements: center line = median; box limits = upper and lower quartiles; whiskers = 1.5 x interquartile range; points = samples.

a

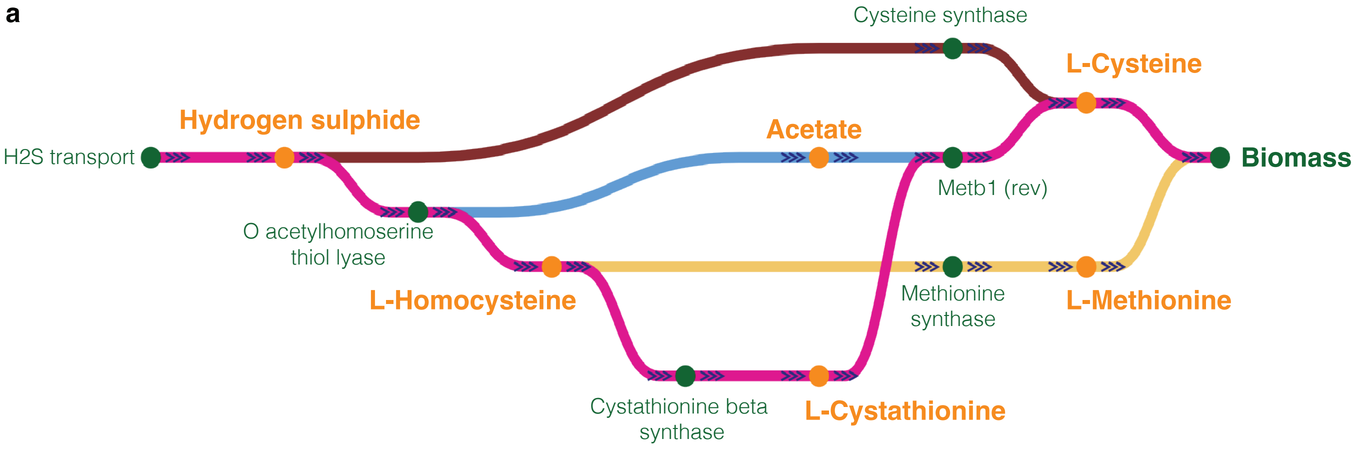

b

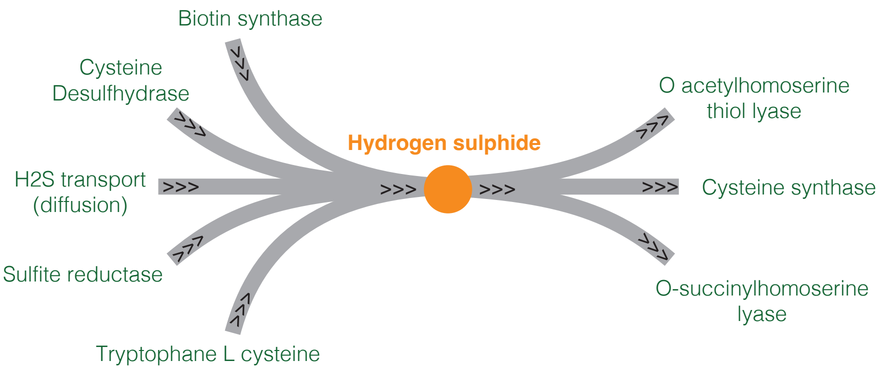

Supplementary Figure S4. Overview of the metabolic reactions utilising imported hydrogen sulphide in *Roseburia intestinalis*, one of the key H_2S consumers that is depleted in the microbiome associated with Crohn’s disease.**a** Shortest *k* paths using H_2S transport as source and biomass reaction as target. **b** All potential reactions leading to production and consumption of H_2S.

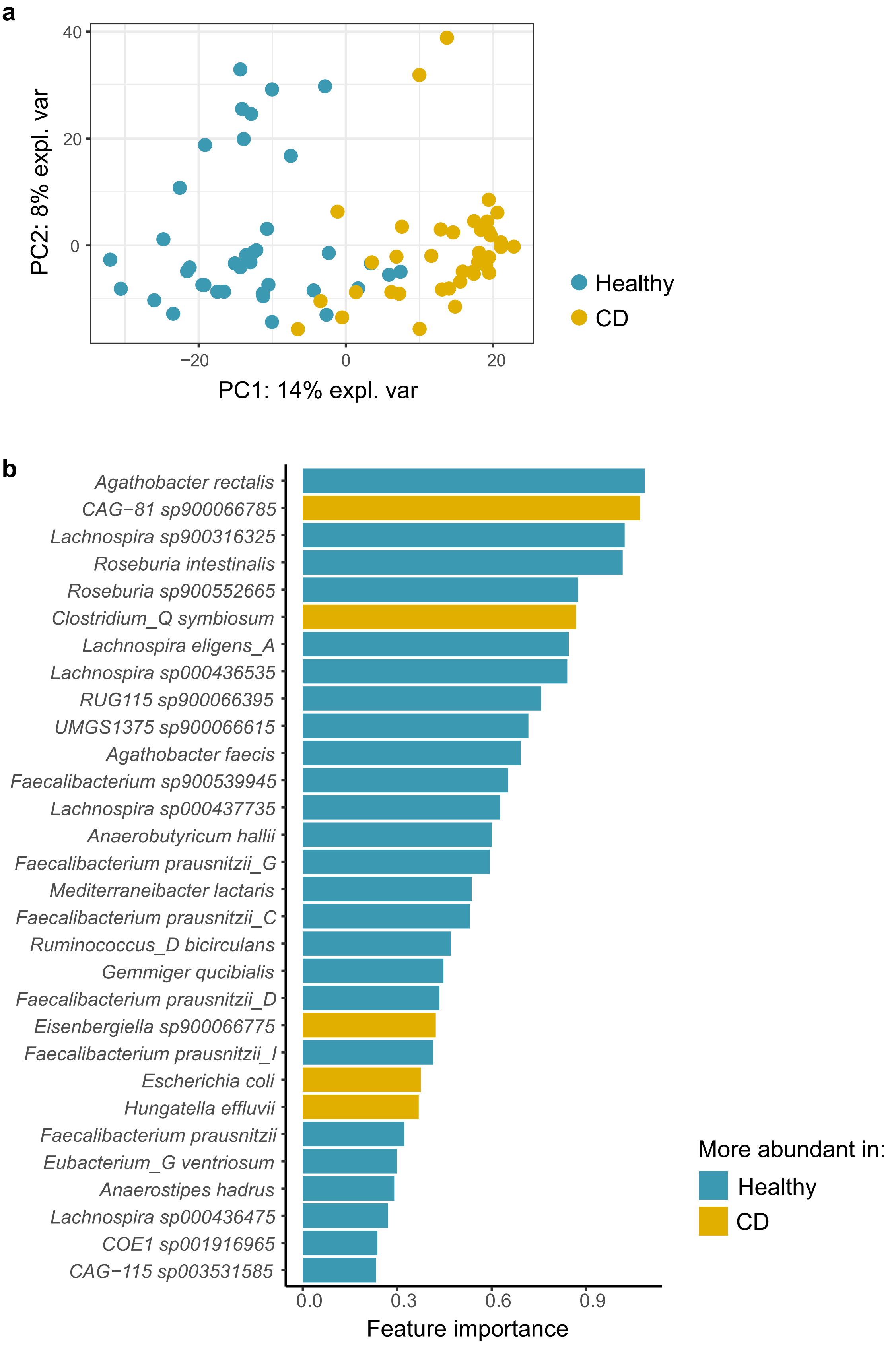

Supplementary figure S5. Beta diversity and machine learning analysis of microbiomes associated with Crohn's disease (CD). **a.** PCA plot of the community beta-diversity (CLR-transformed data). **b.** Random forest feature importance (mean decrease Gini) highlighting which species contribute most to the variance between healthy- and CD- associated microbiomes (30 species with the highest mean decrease Gini shown). Sample size (n) =76 metagenomes (38 in each category, same samples used in the analyses represented in Figure 4e).

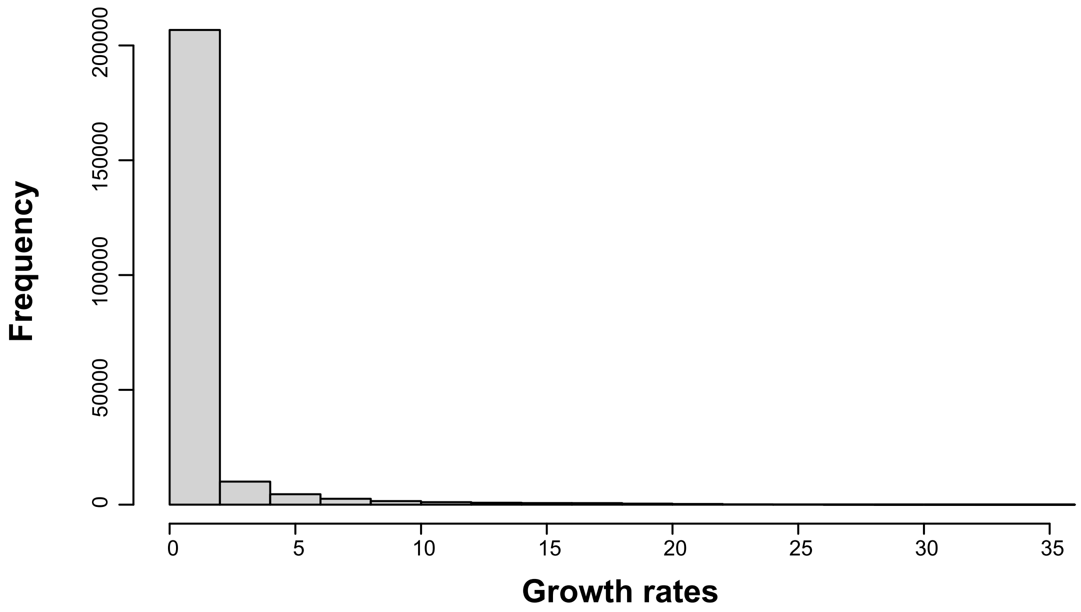

Supplementary Figure S6. Histogram of estimated bacterial growth rates (mmol gDW-1 h-1) in the microbiome communities of 1661 individuals.
