# Supplementary material for: Disease-specific loss of microbial cross-feeding interactions in the human gut
Source: Nat Commun. 2023 Oct 20;14:6546. doi: 10.1038/s41467-023-42112-w (PMC10589287; doi:10.1038/s41467-023-42112-w)
Supplement: Supplementary file 2 — Reporting Summary [file 41467_2023_42112_MOESM2_ESM.pdf]

nature portfolio

Corresponding author(s): Vanessa R. Marcelino, Samuel C. Forster

Last updated by author(s): 2023-Sep-27

Reporting Summary

Nature Portfolio wishes to improve the reproducibility of the work that we publish. This form provides structure for consistency and transparency in reporting. For further information on Nature Portfolio policies, see our [Editorial Policies](#) and the [Editorial Policy Checklist](#).

Statistics

For all statistical analyses, confirm that the following items are present in the figure legend, table legend, main text, or Methods section.

n/a	Confirmed	
☐	☒	The exact sample size (n) for each experimental group/condition, given as a discrete number and unit of measurement
☐	☒	A statement on whether measurements were taken from distinct samples or whether the same sample was measured repeatedly
☐	☒	The statistical test(s) used AND whether they are one- or two-sided<br>*Only common tests should be described solely by name; describe more complex techniques in the Methods section.*
☐	☒	A description of all covariates tested
☐	☒	A description of any assumptions or corrections, such as tests of normality and adjustment for multiple comparisons
☐	☒	A full description of the statistical parameters including central tendency (e.g. means) or other basic estimates (e.g. regression coefficient) AND variation (e.g. standard deviation) or associated estimates of uncertainty (e.g. confidence intervals)
☐	☒	For null hypothesis testing, the test statistic (e.g. F, t, r) with confidence intervals, effect sizes, degrees of freedom and P value noted<br>*Give P values as exact values whenever suitable.*
☒	☐	For Bayesian analysis, information on the choice of priors and Markov chain Monte Carlo settings
☒	☐	For hierarchical and complex designs, identification of the appropriate level for tests and full reporting of outcomes
☒	☐	Estimates of effect sizes (e.g. Cohen's d, Pearson's r), indicating how they were calculated

Our web collection on statistics for biologists contains articles on many of the points above.

Software and code

Policy information about availability of computer code

Data collection	Not applicable as this study did not collect new data.
Data analysis	Quality control was performed with TrimGalore v.0.6.6, seqtk v.1.3 and bowtie v.2.3.5. Assembly and binning was performed with MegaHit v.1.2.9 and Vamb v.3.0.221. QC of the MAGs and classification were performed with CheckM and GTDBtk v.1.5.1. Abundance of MAGs across samples was estimated with KMA v.1.3.13. GEMs were reconstructed with CarveMe v1.5, community-wide modelling was performed with MICOM v.0.26. Calculation of MES and statistical tests were performed with scripts available in Zenodo: https://zenodo.org/record/8223163 (repository v.1.2.2).

For manuscripts utilizing custom algorithms or software that are central to the research but not yet described in published literature, software must be made available to editors and reviewers. We strongly encourage code deposition in a community repository (e.g. GitHub). See the Nature Portfolio guidelines for submitting code & software for further information.

Data

Policy information about availability of data

All manuscripts must include a data availability statement. This statement should provide the following information, where applicable:

- Accession codes, unique identifiers, or web links for publicly available datasets
- A description of any restrictions on data availability
- For clinical datasets or third party data, please ensure that the statement adheres to our policy

The data used in this study is publicly available in the European Nucleotide Archive (ENA). All assemblies and MAGs reconstructed in this study have been deposited

in ENA under project PRJEB63093 [https://www.ebi.ac.uk/ena/browser/view/PRJEB63093]. BioSample IDs for the raw sequence data and assembly IDs for the assemblies performed in this study are provided in Supplementary Data 1. ENA sample accessions for all metagenome bins reconstructed in this study are provided in Supplementary Data 12, and the ENA analysis ID for the 955 species-level MAGs are provided in Supplementary Data 2. All high-quality MAGs are also available in Zenodo24 [https://zenodo.org/record/8223163]. Metabolite classes were inferred from the Human Metabolome Database HMDB 4.0 [https://hmdb.ca].

Human research participants

Policy information about studies involving human research participants and Sex and Gender in Research.

Reporting on sex and gender	Our study cohort included samples from males (n=738), females (n=705) and samples where this information was not provided (254).
Population characteristics	Our samples included healthy and non-healthy individuals from 15 countries. All individuals were over 10 years of age. Our samples include individuals where age was not reported but the study stated that they were collected from adults.
Recruitment	This is a meta-analyses of 33 peer-reviewed studies, each study had their own recruitment protocol.
Ethics oversight	Not applicable - all data used here is publicly available.

Note that full information on the approval of the study protocol must also be provided in the manuscript.

Field-specific reporting

Please select the one below that is the best fit for your research. If you are not sure, read the appropriate sections before making your selection.

☒ Life sciences ☐ Behavioural & social sciences ☐ Ecological, evolutionary & environmental sciences

For a reference copy of the document with all sections, see nature.com/documents/nr-reporting-summary-flat.pdf

Life sciences study design

All studies must disclose on these points even when the disclosure is negative.

Sample size	Sample-size calculation was not performed before the computational analyses. Our initial survey identified 6634 potential samples, which would not be computationally feasible to analyze. Therefore we performed a strict quality control, selecting only samples with very high sequencing depth (15M PE reads after quality control) and selecting a limited number of samples (max=100) per disease category from each study. The fact that we found many significant microbiome-disease associations (accounting for multiple comparisons) indicates that the sample size is sufficient.
Data exclusions	Studies focusing on dietary interventions, medications, exercise and children were excluded. Metagenomes that were not sequenced with Illumina's HiSeq or NovaSeq platforms were also excluded. Samples classified as disease controls and where the health status could not be determined were excluded. Samples from individuals with colorectal adenoma (non-cancerous tumor) and impaired glucose tolerance (pre-diabetes) were excluded, and only individuals with a Body Mass Index (BMI) between 18.5 and 24.9 were included in the healthy cohort. Samples with less than 15M PE reads after quality control were excluded to minimize the impact of sequencing depth. A maximum of 100 samples per disease category from each study were used.
Replication	Our conceptual approach was applied to multiple disease phenotypes. The Crohn's disease results will benefit from analyses of different cohorts in future studies. This was not performed here because our approach relies on MAGs that were generated by co-binning a large, high-quality dataset. The number and completeness of MAGs assembled from an individual study will be limited and therefore would not constitute a replicate.
Randomization	Not applicable to our analyses as this is a cross-sectional study based on a large-scale analysis of publicly available metagenomes.
Blinding	Not applicable to our analyses - information on the health status of the individuals were required to identify the cross-feeding interactions most affected in disease. Patients were not identifiable.

Reporting for specific materials, systems and methods

We require information from authors about some types of materials, experimental systems and methods used in many studies. Here, indicate whether each material, system or method listed is relevant to your study. If you are not sure if a list item applies to your research, read the appropriate section before selecting a response.

Materials & experimental systems

n/a	Involved in the study
☒	☐ Antibodies
☒	☐ Eukaryotic cell lines
☒	☐ Palaeontology and archaeology
☒	☐ Animals and other organisms
☒	☐ Clinical data
☒	☐ Dual use research of concern

Methods

n/a	Involved in the study
☒	☐ ChIP-seq
☒	☐ Flow cytometry
☒	☐ MRI-based neuroimaging
