# Supplementary material for: Disease-specific loss of microbial cross-feeding interactions in the human gut
Source: Nat Commun. 2023 Oct 20;14:6546. doi: 10.1038/s41467-023-42112-w (PMC10589287; doi:10.1038/s41467-023-42112-w)
Supplement: Supplementary file 4 — Description of Additional Supplementary Files [file 41467_2023_42112_MOESM4_ESM.pdf]

Description of Additional Supplementary Files

File Name: Supplementary Data 1
Description: Metagenome samples, studies and corresponding metadata utilised to perform a meta-analysis of human gut metagenomes.

File Name: Supplementary Data 2
Description: 955 species-level metagenome-assembled genomes and their taxonomic classification. The list contains one representative MAG per species, defined at 95% ANI.

File Name: Supplementary Data 3
Description: Metagenome-assembled genomes identified exclusively in diseased individuals (a) or healthy individuals (b).

File Name: Supplementary Data 4
Description: MESs for each metabolite in the microbiomes of associated with type 2 diabetes, when compared to healthy microbiomes. These analyses were performed using a one-sided Kruskal-Wallis test, and significant values adjusted for multiple comparisons (i.e. $p < 0.0001373626$ were considered significant).

File Name: Supplementary Data 5
Description: The relationship between species diversity and number of producers or consumers. A linear model was used to test for differences in the slope of the correlation between species diversity and number of producers or consumers. These analyses were performed with the R function lm(number_prod_or_cons ~ species_diversity*category_prod_cons). Significant differences between slopes were tested with a two-sided t-test, and corrected for multiple comparisons with the Bonferroni method ($p < 0.0001113586$). "consumers more affected" indicates that the slope of the consumer x spp diversity correlation was significantly steeper than the producer x spp diversity correlation slope. "producers more affected" indicates that the slope of the producer x spp diversity correlation was significantly steeper than the consumer slope. See figure 3 b-p for examples of these slopes. Metabolites present in less than 50 microbiome samples were excluded from the analyses.

File Name: Supplementary Data 6
Description: Differences in MES scores between healthy and non-healthy microbiomes from a Crohn's disease study (He et al. GigaScience 2017). Differences were accessed with a Kruskal-Wallis test, using the Bonferroni method to account for multiple comparisons (p considered significant if < 0.0001396648, indicated with '*'). MES = Metabolite Exchange Score; CD = Crohn's disease; std = Standard deviation.

File Name: Supplementary Data 7
Description: Differences in diversity and total flux of potential H_2S producers and consumers. Fluxes were calculated in millimoles per gram [dry weight {DW}] per hour, multiplied by species abundance (absolute counts from rarefied datasets).

File Name: Supplementary Data 8
Description: Confounder tests to assess whether the number of H_2S producers or consumers is different in health and Crohn's disease microbiomes after accounting for species diversity.

File Name: Supplementary Data 9
Description: Survey of genes involved in H2S cycling in the MAGs observed the He et al (Gigascience 2017) case-control Crohn's disease study.

File Name: Supplementary Data 10
Description: Test for differential distribution of genes involved in the metabolism of hydrogen sulphide between healthy and diseased microbiomes. These analyses were performed with a linear model, using species diversity as an independent variable. Asterisks indicate a significant difference (p-value of the associated two-sided t-test) after Bonferroni correction (i.e. $p<0.00125$)

File Name: Supplementary Data 11
Description: Species responsible for production and consumption of H2S within the microbiome associated with Crohn's disease (CD) and healthy controls. MAG = Metagenome-assembled genome. Category = based on the net flux of all samples within the healthy or disease groups. Weighted flux sum = sum of (flux × relative abundance) across samples within healthy and CD cohorts. Top 10 H2S consumers with reduced weighted flux sum in CD are highlighted in blue. Top 10 H2S producers with increased weighted flux sum in CD are highlighted in red.

File Name: Supplementary Data 12
Description: List of metagenome bins reconstructed in this study and their corresponding ENA sample accession IDs.
